# Frequent Seizures Are Associated with a Network of Gray Matter Atrophy in Temporal Lobe Epilepsy with or without Hippocampal Sclerosis

**DOI:** 10.1371/journal.pone.0085843

**Published:** 2014-01-27

**Authors:** Ana C. Coan, Brunno M. Campos, Clarissa L. Yasuda, Bruno Y. Kubota, Felipe PG. Bergo, Carlos AM. Guerreiro, Fernando Cendes

**Affiliations:** Neuroimaging Laboratory, Department of Neurology, State University of Campinas, Campinas, SP, Brazil; University of Ulm, Germany

## Abstract

**Objective:**

Patients with temporal lobe epilepsy (TLE) with hippocampal sclerosis (HS) have diffuse subtle gray matter (GM) atrophy detectable by MRI quantification analyses. However, it is not clear whether the etiology and seizure frequency are associated with this atrophy. We aimed to evaluate the occurrence of GM atrophy and the influence of seizure frequency in patients with TLE and either normal MRI (TLE-NL) or MRI signs of HS (TLE-HS).

**Methods:**

We evaluated a group of 172 consecutive patients with unilateral TLE-HS or TLE-NL as defined by hippocampal volumetry and signal quantification (122 TLE-HS and 50 TLE-NL) plus a group of 82 healthy individuals. Voxel-based morphometry was performed with VBM8/SPM8 in 3T MRIs. Patients with up to three complex partial seizures and no generalized tonic-clonic seizures in the previous year were considered to have infrequent seizures. Those who did not fulfill these criteria were considered to have frequent seizures.

**Results:**

Patients with TLE-HS had more pronounced GM atrophy, including the ipsilateral mesial temporal structures, temporal lobe, bilateral thalami and pre/post-central gyri. Patients with TLE-NL had more subtle GM atrophy, including the ipsilateral orbitofrontal cortex, bilateral thalami and pre/post-central gyri. Both TLE-HS and TLE-NL showed increased GM volume in the contralateral pons. TLE-HS patients with frequent seizures had more pronounced GM atrophy in extra-temporal regions than TLE-HS with infrequent seizures. Patients with TLE-NL and infrequent seizures had no detectable GM atrophy. In both TLE-HS and TLE-NL, the duration of epilepsy correlated with GM atrophy in extra-hippocampal regions.

**Conclusion:**

Although a diffuse network GM atrophy occurs in both TLE-HS and TLE-NL, this is strikingly more evident in TLE-HS and in patients with frequent seizures. These findings suggest that neocortical atrophy in TLE is related to the ongoing seizures and epilepsy duration, while thalamic atrophy is more probably related to the original epileptogenic process.

## Introduction

The structural damage in patients with temporal lobe epilepsy (TLE) extends beyond the mesial temporal structures [1]. This knowledge corroborates the hypothesis that TLE is in fact a network disease [2]. However, the specific causes for the extra-hippocampal atrophy in TLE are not well understood.

Among patients with TLE, the phenotypic presentation, natural history and response to treatment vary significantly. Although 60–70% of cases of TLE show MRI signs of hippocampal sclerosis (HS), a significant number of patients have normal MRIs [3]. Surgical specimens from drug-resistant MRI-negative TLE rarely show hippocampal cell loss compatible with HS [4], [5], and this is considered a distinct entity from TLE-HS^5^.

Although TLE associated with MRI signs of HS is the prototype of refractory epilepsy, there are also patients with good seizure control [6], [7] or who are seizure-free [8]. In TLE with normal MRI, it is still a mystery why some patients are refractory to antiepileptic-drugs (AEDs) [6]. If the degree of initial damage is the only factor associated with refractoriness to AEDs and ongoing seizures cause additional damage, why these patients do not show clear MRI abnormalities?

In this study we aimed to evaluate the occurrence of gray matter (GM) atrophy in patients with TLE and normal MRI (TLE-NL) and TLE-HS. We hypothesized that in patients with TLE and an appropriately defined absence of MRI signs of HS the network of structural damage might differ from those with HS. As a second hypothesis, we considered that differences in the frequency of seizures under AED treatment and the duration of epilepsy might also influence the patterns of structural damage in these patients.

## Methods

### Ethical aspects

The Ethics Committee of UNICAMP approved the present study. All patients signed a written informed consent form approved by the Ethics Committee of UNICAMP before enrolling in the study.

### Patient's selection and clinical data

We evaluated a group of 172 consecutive patients with diagnosis of unilateral TLE-HS and TLE-NL as defined by hippocampal volumetry and signal quantification (122 TLE-HS and 50 TLE-NL) [9].

All patients had the epileptogenic zone (EZ) lateralized by ictal and/or inter-ictal scalp EEG (10–20 system, including zygomatic electrodes). Although not all patients had ictal scalp EEG, they all were evaluated with repeated and prolonged wakefulness and sleep scalp EEG (average time 200 minutes; range 80 to 360 minutes). Patients with bitemporal ictal onset were not included. Only patients with unilateral temporal discharges on inter-ictal scalp EEGs or at least 80% of the inter-ictal discharges localized in one of the temporal lobes were selected. Patients with the EZ contralateral to the MRI signs of HS were also not included. In order to decrease the possibility of neocortical epilepsy, only those with inter-ictal epileptiform discharges characterized by isolated anterior temporal spikes either followed or not by slow waves were selected. Those with spikes localized in the posterior temporal lobe, polyspikes or secondary bisynchronous discharges were not included.

Family history was defined as at least one first or second-degree family member with any type of epilepsy. Duration of epilepsy was defined as the age at MRI acquisition minus the age of epilepsy onset, while the time of active epilepsy was defined as the age at MRI acquisition minus the age of epilepsy onset minus the sum of periods of seizure remission longer than two years. Patients with up to three complex partial seizures (CPS) and no generalized tonic-clonic seizures (GTCS) in the previous year were considered to have infrequent seizures. Those who did not fulfill these criteria were considered to have frequent seizures. Patients who were seizure free for at least two years were considered in remission.

### MRI acquisition and GM atrophy analysis

To detect GM atrophy, automated analysis of the brain structure as a whole was performed with VBM. VBM consists of an automatic image analysis that allows comparison of the local intensities of brain tissues in groups of individuals without the need for prior definition of a region of interest [10]. The VBM8/SPM8 toolbox was used (Welcome Department of Cognitive Neurology, http://www.fil.ion.ucl.ac.uk) with 3D sagittal T1-weighted images (voxel size  = 1×1×1 mm^3^, no gap, TR = 7 ms, TE = 3.2 ms, flip angle  = 8°, matrix  = 240×240; FOV = 240×240) acquired in a 3T MRI scanner (Philips Medical Systems, Best, The Netherlands). Images from patients with the EZ on the right side were flipped in left-right orientation so the EZ of all patients was aligned on the left. A control group of 82 healthy subjects (age and sex matched with patients) was used for comparison (60% female, median age 40 years, range 21–70), with a proportion of the controls comparable with each patient group also flipped in the right-left orientation. In order to account for the age range, age was used as a covariate in the statistical model.

After VBM preprocessing, the MRI homogeneity and co-registration test detected 10 outliers: three controls, four TLE-HS and three TLE-NL. These individuals were therefore excluded from the final analysis and the preprocessing steps were redone. The imaging preprocesses steps included spatial normalization to the same stereotaxic space (MNI-152 template), modulation (to correct for possible volume changes due to normalization and allow evaluation of abnormal volumes) [11], [12], and segmentation into different tissues, including GM. The DARTEL algorithm was used in the pre-processing steps in order to increase the accuracy of the alignment between subjects [13]. The resultant GM images were smoothed with an 8-mm full width at half maximum isotropic Gaussian kernel.

The GM post-processed images of both groups were compared using a voxel-wise statistical analysis looking for the areas of volume reduction or increase in patients. Two sample T-tests with an initial statistical threshold of p<0.001, uncorrected and a minimum cluster size of 30 contiguous voxels were used. As a second step, the results were corrected with a more stringent statistical threshold of p<0.05 family wise error (FWE) corrected.

#### GM atrophy: seizure frequency and correlation with epilepsy duration

For a secondary analysis, patients were divided into four subgroups according to the seizure frequency: i) TLE-NL with infrequent seizures; ii) TLE-NL with frequent seizures; iii) TLE-HS with infrequent seizures; iv) TLE-HS with frequent seizures. In order to avoid differences in clinical data [14]–[16] that might contribute to the pattern of GM atrophy in TLE, such as the side with EZ [16], [17], duration of epilepsy [17], [18] or family history [19], these four groups were paired according to their clinical characteristics. For this purpose, the subgroup with the smallest number of patients was considered the model and an equal number of patients from all the other sub-groups were chosen in consecutive order until similar characteristics were achieved. Two-sample T-tests for each patient subgroup versus controls were done with an initial statistical threshold of p<0.001, uncorrected and a minimum cluster size of 30 contiguous voxels. As a second step, the results were corrected with a threshold of p<0.05 FWE corrected.

The GM volume was also correlated with the duration of epilepsy in patients with TLE-HS and TLE-NL (multiple regression with initial statistical threshold of p<0.001, uncorrected and a minimum cluster size of 30 contiguous voxels plus, as a second step, additional correction with a threshold of p<0.05 FWE corrected).

## Results

### Clinical characteristics of TLE patients

The VBM analysis included 165 patients: 118 TLE-HS and 47 TLE-NL. The clinical data is summarized in Table 1.

**Table 1:** Demographic and clinical data of TLE-HS and TLE-NL patients.

	TLE-HS (n = 118)	TLE-NL (n = 47)	P value
Sex	74 (63%) female; 44 (37%) male	27 (57%) female; 20 (43%) male	*X* ^2^, p = 0.531
Age (range)	46 years (17–73)	43 years (19–74)	T-test, p = 0.228
Age of seizure onset (range)	12 years (0–50)	16 years (2–45)	**T-test, p = 0.004**
Family history of epilepsy	40 (34%)	27 (57%)	*X* ^2^, **p = 0.006**
FS/IPI	13(11%)/26(22%)	3(6%)/12(25%)	*X* ^2^, p = 0.356/p = 0.650
SE	3 (2%)	1 (2%)	Fischeŕs exact test, p = 0.858
Duration of epilepsy (range)	32 years (2–62)	23 years (3–50)	**T-test, p = 0.001**
Time of active epilepsy (range)	28 years (2–62)	22 years (3–47)	**T-test, p = 0.008**
Seizure frequency	31 (26%) infrequent seizures; 87 (74%) frequent seizures	16 (34%) infrequent seizures; 31 (66%) frequent seizures	*X* ^2^, p = 0.381
Seizure remission	12 (10%)	10 (21%)	*X* ^2^, p = 0.063
Laterality of epileptogenic zone	64 left (54%); 54 right (46%)	37 left (79%); 10 right (21%)	*X* ^2^, **p = 0.001**
Number of patients with SGTCS in the previous year	14 (12%)	12 (25%)	*X* ^2^, **p = 0.03**

TLE-HS: temporal lobe epilepsy with MRI signs of hippocampal sclerosis; TLE-NL: temporal lobe epilepsy with normal MRI; FS: febrile seizure; IPI: initial precipitating injury; SE: status epilepticus; SGTCS: secondary generalized tonic-clonic seizures.

Sixty-one patients (36%) had unilateral temporal ictal EEG recordings; among the remaining patients, 67 (60%) had exclusively unilateral inter-ictal temporal discharges and 44 (40%) had bitemporal inter-ictal temporal discharges, with at least 80% of these in one temporal lobe.

Age of epilepsy onset was greater in patients with TLE-NL (two sample T-test, p = 0.004). Duration of epilepsy and time of active epilepsy were longer in patients with TLE-HS (two sample T-test, p = 0.001 and p = 0.008, respectively). Family history of epilepsy was more frequent in patients with TLE-NL (*X* ^2^, p = 0.006). TLE-NL patients had a higher incidence of patients with SGTCS (*X* ^2^, p = 0.03). The median number of different AEDs tried since the onset of epilepsy was five in the TLE-HS group (ranging from two to ten) and four in the TLE-NL group (range two to nine).

### Gray matter atrophy: TLE-HS versus TLE-NL

Patients with TLE-HS had diffuse GM atrophy, including the ipsilateral mesial temporal structures, superior and middle portion of the temporal lobe, post-central gyrus, middle occipital gyrus, precuneus and caudate, bilateral thalami, and cuneus and contralateral pre-central gyrus (Table S1) (two-sample T-test, p<0.05, FWE corrected, minimum cluster of 30 voxels). In patients with TLE-NL, VBM analysis did not survive the FWE correction. However, GM atrophy was observed with a statistical threshold of p<0.001 uncorrected (minimum cluster size of 30 contiguous voxels). In this group, GM atrophy was observed in the ipsilateral orbitofrontal cortex and post-central gyrus, bilateral thalami, contralateral pre-central gyrus, cuneus and middle occipital gyrus (Table S1).

**Figure 1:**
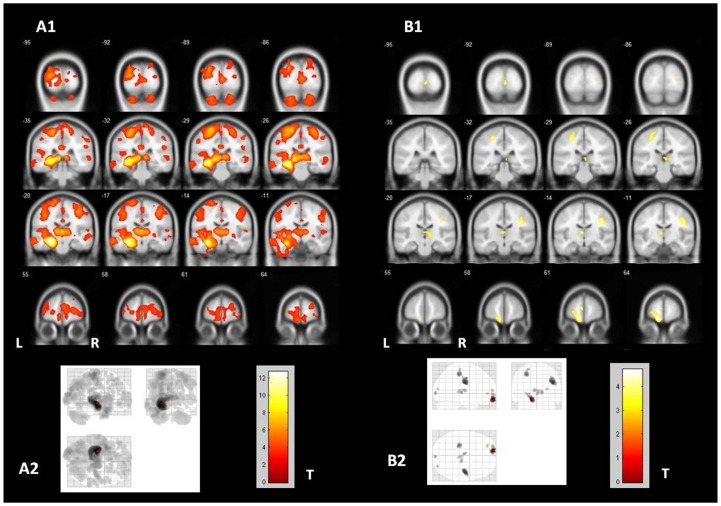
Gray matter atrophy in TLE-HS and TLE-NL. VBM demonstrated significant areas of diffuse gray matter volume loss in TLE-HS and TLE-NL. A1 and A2 (“glass view”) show the areas of gray matter atrophy in TLE-HS (two-sample T-test, p<0.001, uncorrected, minimum threshold cluster of 30 voxels); B1 and B2 (“glass view”) show the areas of gray matter atrophy in TLE-NL (two-sample T-test, p<0.001, uncorrected, minimum threshold cluster of 30 voxels). TLE-HS: temporal lobe epilepsy with MRI signs of hippocampal sclerosis; TLE-NL: temporal lobe epilepsy with normal MRI; VBM: voxel based morphometry; T: t-value; L: left; R: right.

**Figure 2:**
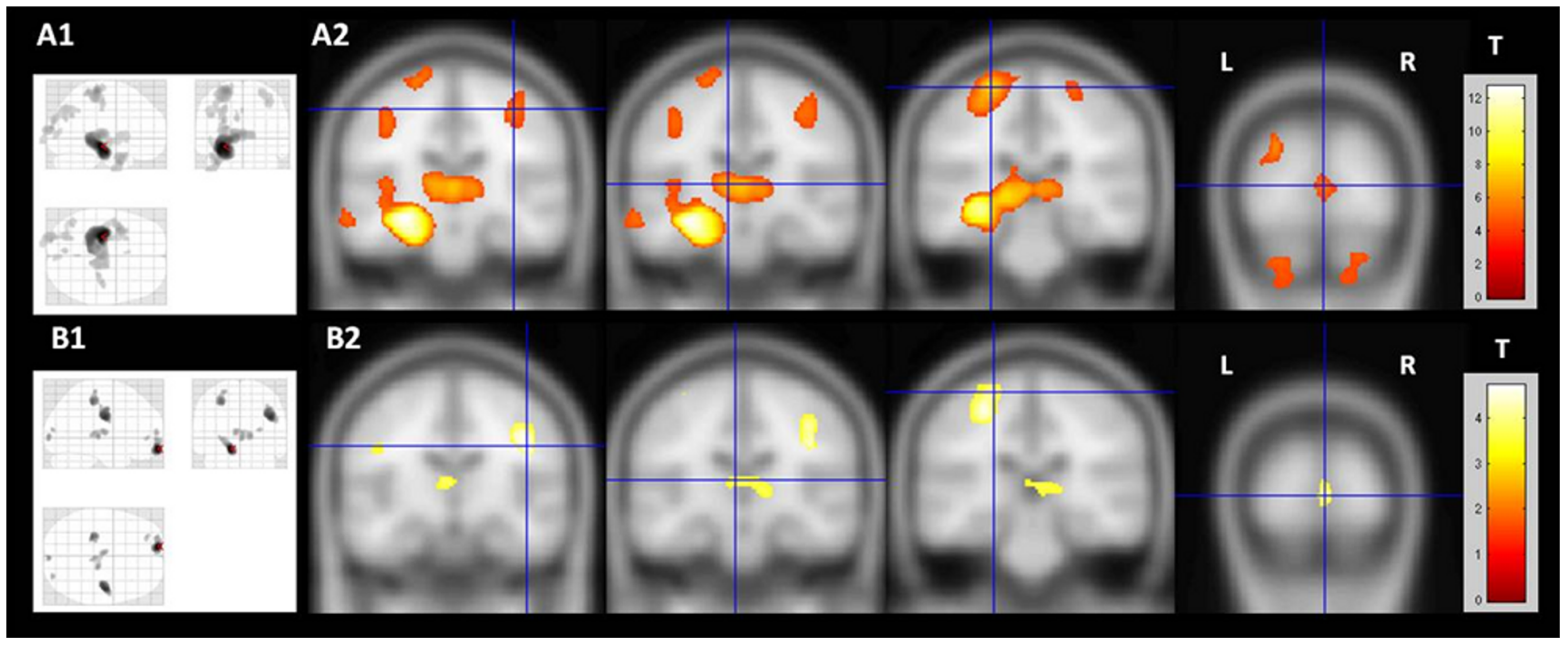
Common areas of gray matter atrophy in TLE-HS and TLE-NL. The slices demonstrate the common brain areas with gray matter atrophy in TLE-HS and TLE-NL. TLE-HS results are shown with a more stringent statistical significance in order to facilitate the comparison. A1 (“glass view”) and A2 show the areas of gray matter atrophy in TLE-HS (two-sample T-test, p<0.05, FWE corrected, minimum threshold cluster of 30 voxels); B1 (“glass view”) and B2 show the areas of gray matter atrophy in TLE-NL (two-sample T-test, p<0.001, uncorrected, minimum threshold cluster of 30 voxels). TLE-HS: temporal lobe epilepsy with MRI signs of hippocampal sclerosis; TLE-NL: temporal lobe epilepsy with normal MRI; VBM: voxel based morphometry; T: t-value; L: left; R: right.

Figure 1 shows GM atrophy detected by VBM analysis in TLE-HS and TLE-NL. For more appropriate comparison, the results for both groups are shown with the same statistical threshold (p<0.001 uncorrected, minimum cluster of 30 voxels). All the clusters of GM atrophy observed in the TLE-NL group were also observed in the TLE-HS group. When the VBM threshold was set to p = 0.05 FWE corrected in the TLE-HS group, the same clusters with atrophy detected in the TLE-NL group were observed, except for the orbitofrontal cortex (Figure 2).

The VBM analysis looking for GM atrophy was also done excluding the three patients with previous history of *status epilepticus*. However, no significant difference was observed in the results after excluding these individuals.

### Gray matter increase: TLE-HS versus TLE-NL

**Figure 3:**
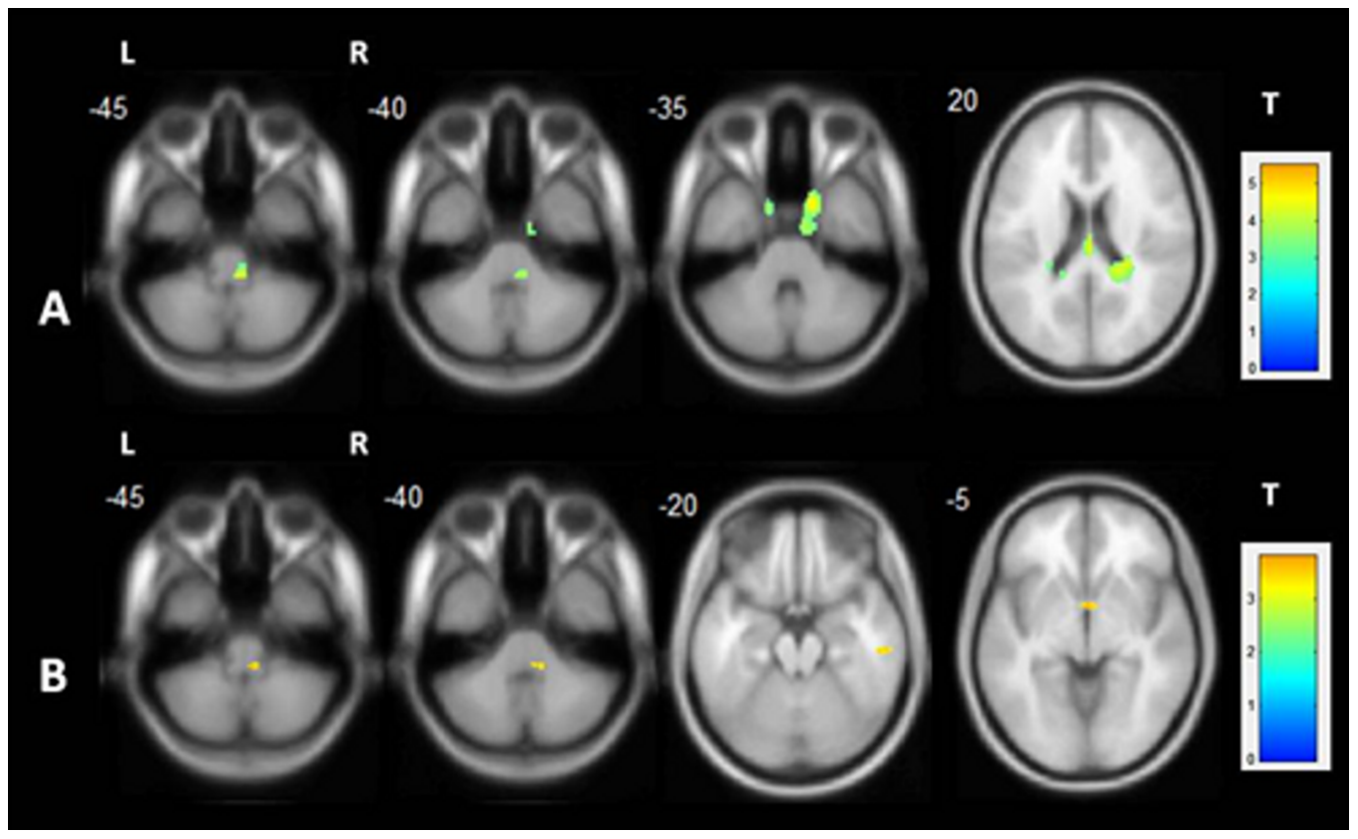
Gray matter volume increase in TLE-HS and TLE-NL. VBM demonstrated areas of gray matter increase in TLE-HS and TLE-NL. A: shows the areas of gray matter volume increase in TLE-HS (two-sample T-test, p<0.001, uncorrected, minimum threshold cluster of 30 voxels); B: shows the areas of gray matter volume increase in TLE-NL (two-sample T-test, p<0.001, uncorrected, minimum threshold cluster of 30 voxels). TLE-HS: temporal lobe epilepsy with MRI signs of hippocampal sclerosis; TLE-NL: temporal lobe epilepsy with normal MRI; VBM: voxel based morphometry; T: t-value; L: left; R: right.

In TLE-HS, GM increase was observed in the ipsilateral uncus, contralateral cingulate gyrus and cerebellum, and in TLE-NL in contralateral inferior temporal gyrus and anterior cingulate (Table S1) (Figure 3). Additionally, both groups had GM volume increase in the contralateral dorsolateral portion of the pons (Two-sample T-tests, p<0.001, uncorrected, minimum of 30 voxels). No clusters of GM volume increase persisted after the additional correction of the T-tests for multiple comparisons (FWE).

### Gray matter atrophy: seizure frequency

Patients were evaluated according to the four subgroups: i) TLE-NL with infrequent seizures; ii) TLE-NL with frequent seizures; iii) TLE-HS with infrequent seizures; iv) TLE-HS with frequent seizures. TLE-NL with infrequent seizures was the group with the smallest number of patients (N = 16) and the other groups were composed as described in the methods. The characteristics of each of the four subgroups are described in Table S2. In these VBM analyses, only the results of the subgroups of patients with TLE-HS survived the statistical threshold of p<0.05, FWE corrected. Therefore, for adequate comparison, all the results described are referent to p<0.001, uncorrected.

**Figure 4:**
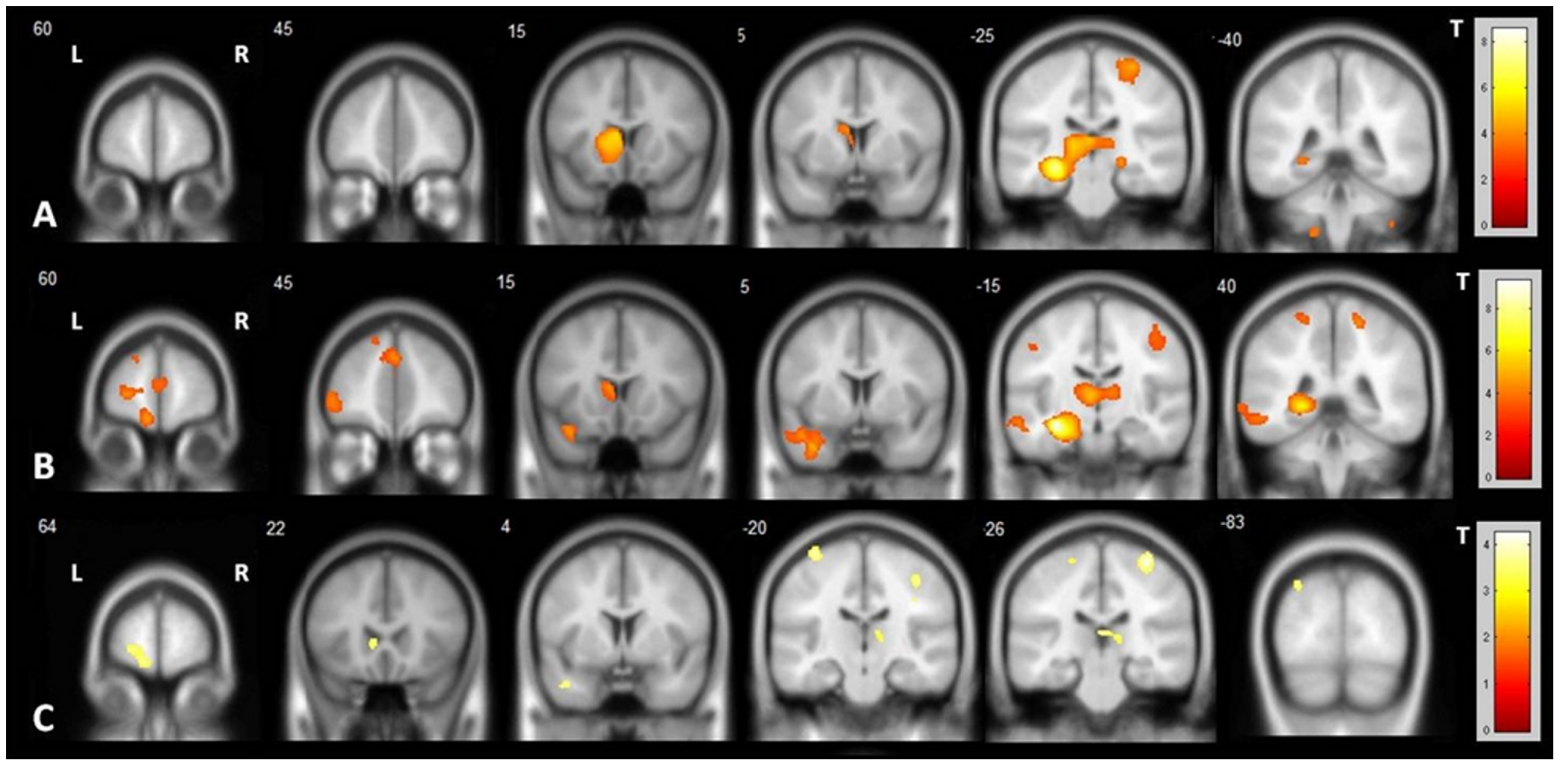
Patterns of gray matter atrophy according to seizure frequency in TLE-HS and TLE-NL. VBM demonstrated significant areas of diffuse gray matter atrophy in TLE-HS patients with infrequent and frequent seizures but only in TLE-NL with frequent seizures. A: areas of gray matter atrophy in TLE-HS with infrequent seizures (two-sample T-test, p<0.001, uncorrected, minimum threshold cluster of 30 voxels); B: areas of gray matter atrophy in TLE-HS with frequent seizures (two-sample T-test, p<0.001, uncorrected, minimum threshold cluster of 30 voxels); C: areas of gray matter atrophy in TLE-NL with frequent seizures (two-sample T-test, p<0.001, uncorrected, minimum threshold cluster of 30 voxels). TLE-HS: temporal lobe epilepsy with MRI signs of hippocampal sclerosis; TLE-NL: temporal lobe epilepsy with normal MRI; VBM: voxel based morphometry; T: t-value; L: left; R: right.

In TLE-HS, in both subgroups of patients with frequent and infrequent seizures, GM atrophy was observed in the ipsilateral mesial temporal structures, caudate, bilateral thalamus and contralateral precentral gyrus. However, distinct from TLE-HS with infrequent seizures, patients with TLE-HS and frequent seizures presented more diffuse GM atrophy including other neocortical structures, mainly in the ipsilateral frontal lobe (Figure 4; Table S3).

In TLE-NL, patients classified as frequent seizures presented GM atrophy in the ipsilateral orbitofrontal cortex, bilateral thalamus and precentral gyrus, while those classified as experiencing infrequent seizures had no detectable GM atrophy (Figure 4; Table S3).

### Gray matter increase: seizure frequency

No GM increase was observed in any of the four TLE subgroups.

### Correlation of duration of epilepsy and GM atrophy

**Figure 5:**
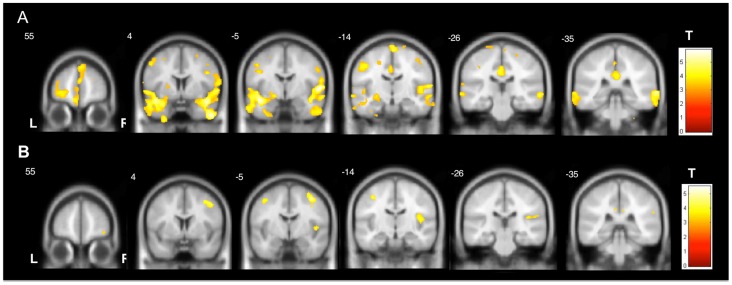
Areas of gray matter atrophy associated with epilepsy duration in TLE-HS and TLE-NL. Both groups of patients had areas of GM atrophy associated with epilepsy duration. In TLE-HS, the duration of epilepsy was correlated with GM atrophy, mainly in the bilateral anterior and lateral temporal lobes (A), while in TLE-NL it was more pronounced in bilateral precentral areas and contralateral insula (B) (Multiple regression, p<0.001, uncorrected, minimum threshold cluster of 30 voxels). TLE-HS: temporal lobe epilepsy with MRI signs of hippocampal sclerosis; TLE-NL: temporal lobe epilepsy with normal MRI; T: t-value; L: left; R: right.

In TLE-HS, the duration of epilepsy was correlated with GM atrophy mainly in the bilateral anterior and lateral temporal lobes, while in TLE-NL it was more pronounced in bilateral frontal regions and contralateral insula (multiple regression, p<0.001, uncorrected, minimum of 30 voxels) (Figure 5; Table S4). In TLE-HS, the GM atrophy in the ipsilateral middle temporal gyrus and contralateral superior temporal gyrus persisted after correction of the T-tests for multiple comparisons (FWE). In TLE-NL, no clusters of GM atrophy persisted after the additional correction of the T-tests for multiple comparisons.

## Discussion

We demonstrated that a network of diffuse GM atrophy occurs in both TLE-HS and TLE-NL and that the atrophy in some regions is common to both groups, although in TLE-NL there is no detectable atrophy in the mesial temporal structures. In TLE-HS, GM atrophy is more pronounced and occurs in patients with frequent as well as infrequent seizures, while in TLE-NL it is only observed in those with frequent seizures. The duration of the epilepsy is also associated with extra-hippocampal atrophy.

Although diffuse GM atrophy has been consistently described in TLE patients [1], [16]–[18], the causes of this structural damage remain unclear. In the present study, from a large group of individuals with TLE with detailed clinical information, we were able not only to demonstrate differences in GM atrophy in patients with and without HS, but also to compose very homogeneous subgroups in order to isolate specific characteristics such as seizure frequency. Moreover, the definition of MRI signs of HS in our group was done by combining visual analysis of MRIs by experts plus hippocampal volumetry and T2 signal quantification. Using this approach, we were able to significantly decrease the odds of assigning patients with subtle signs of HS not detectable by visual analysis in the TLE-NL group, which was corroborated by the absence of mesial structure atrophy in the VBM analysis. In fact, surgical series of AED resistant TLE-NL defined in modern MRI protocols demonstrate that HS histopathology is found in only a small percentage of the patients [4], [5]. Studies that can better characterize these individuals might help our understanding of the differences between TLE-HS and TLE-NL as well as the pathophysiology of MRI-negative patients.

### Areas of GM atrophy in TLE-HS and TLE-NL

Although VBM analysis demonstrated diffuse GM atrophy that was strikingly more evident in TLE-HS, we were able to demonstrate that extra-temporal GM atrophy also occurs in TLE-NL. GM atrophy in extra-hippocampal regions has been consistently reported in previous investigations of TLE-HS patients [1], [12], [17], [18]; however, for TLE-NL this is a matter of debate, with different results in previous studies [16], [20], [21]. VBM analysis demonstrated regions of GM atrophy that were common in TLE-HS and TLE-NL, and these were localized outside the temporal lobes. In fact, the areas of extra-temporal atrophy detected in TLE-HS with a more stringent statistical threshold were similar to those detected in TLE-NL in a less robust statistical analysis.

Two regions of extra-temporal GM atrophy were consistently evident in all our VBM analyses: thalamus and sensorimotor cortex. Bilateral thalamic atrophy has been repeatedly described in TLE-HS in VBM studies [22], [23] as well as in manual volumetry reports [24]. This atrophy has predominance in the anterior group or dorsomedial nucleus [25], [26], confirming the increased damage in nuclei with connections to limbic pathways. In our results, although TLE-NL patients had no detectable atrophy in the mesial temporal structures, a pattern of thalamic atrophy similar to the TLE-HS group was observed. This may be indirect evidence that the thalamic atrophy could be secondary to an epileptic network connecting the anterior thalamus and the mesial temporal lobe, also in patients with TLE-NL. In fact, evidence of seizure onset in the hippocampus of TLE-NL patients has been reported based on intracranial EEG studies despite the absence of HS findings in histology [4]. We hypothesized that the hippocampus and other mesial structures are part of the epileptic network of TLE-NL, although no atrophy or cellular loss are detected in these regions. This is concordant with the knowledge that epileptic networks in patients with TLE comprise a complex group of areas with structural damage and/or functional abnormalities that interact pathologically to determine ictal and interictal behavior in each individual [2].

Structural damage in the pre/postcentral cortex has also been described in other MRI quantification studies of TLE-HS [18], [23], [27], [28] and TLE-NL [15], [28], [29]. Possible causes of the atrophy detected in these regions are neuronal loss secondary to the excitotoxicity of seizure spread [30], [15] and the duration of the epilepsy [18], [31]. Hyperperfusion in the ipsilateral precentral and contralateral postcentral gyrus has also been demonstrated by ictal SPECT studies of TLE, which corroborates the hypothesis of seizure propagation to these areas [32]. In our study, pre/postcentral cortex atrophy was detected in TLE-HS and TLE-NL with frequent seizures, but also in TLE-HS with infrequent seizures. These findings do not contradict the hypothesis of seizure burden as a possible cause of atrophy in the sensorimotor cortex, because the exact quantification of number of seizures since the onset of epilepsy is not possible. Moreover, while the atrophy in the pre/postcentral cortex was bilateral in the group with frequent seizures, in TLE-HS with infrequent seizures it was only observed contralateral to the EZ and in a smaller cluster of voxels.

### Areas of GM increase in TLE-HS and TLE-NL

Areas of GM volume increase are less often reported in TLE and are mostly noted in patients with epilepsy and suspected focal cortical dysplasias (FCD) [33]. Similar to our results, GM increase in VBM studies of TLE is more often described in the temporal lobes [22], [34]. Although it can represent subtle FCDs that may be observed in the neocortical temporal tissues of surgical specimens of TLE patients [35], at the moment there is no study correlating these VBM findings and histopathology.

We also observed increased GM in the dorsolateral portion of the pons contralateral to the EZ in both TLE-HS and TLE-NL. The participation of the pons in the network of TLE has not been consistently evaluated. Bilateral hypermetabolism of this structure has been demonstrated in the post-ictal phase in SPECT studies [36] and, more recently, decreased functional connectivity between the left amygdala and the bilateral paramedian pontine area in TLE patients was observed [37]. The dorsal pons is connected to the hippocampus through the locus coeruleus nucleus, providing the source of noradrenaline to the hippocampal neurons and probably participating in memory formation [38]. Due to the small size of the pons and the limitation of the present method in identifying specific regions of the brainstem, a consistent hypothesis regarding the involvement of the pons in the structural network of TLE cannot be formulated. Further studies with more appropriate techniques are encouraged.

### Differences in GM atrophy in patients with frequent or infrequent seizures and duration of epilepsy

In this study, we aimed to evaluate the role of HS and seizure frequency in the diffuse GM atrophy observed in TLE. Distinct from previous reports, we were able to pair in frequent and infrequent seizures groups all the characteristics previously reported in the literature that might influence the pattern of GM atrophy in TLE, including age of seizure onset, duration of epilepsy, time of active epilepsy, family history of epilepsy, history of febrile convulsion and IPI, and occurrence of SGTCS [17]–[19]. Although the selection of patients for the subgroups may seem arbitrary, we also compared the patterns of GM atrophy in these subgroups including all patients irrespective of their clinical data and the results were similar to the ones described here (data not shown).

With this secondary analysis we could observe that the presence of HS is consistent with more pronounced GM atrophy. Also, we demonstrated that although patients with TLE-HS and infrequent seizures have diffuse GM atrophy, this is more restricted to areas with connections to the mesial temporal structures, as the anterior thalamus. Other distant regions such as the frontal and parietal cortex presented less diffuse damage in TLE-HS with infrequent seizures. In contrast, GM atrophy was not observed in TLE-NL patients with infrequent seizures but it occurred in the group of TLE-NL with frequent seizures.

One important aspect that has been described as associated with GM damage in patients with refractory TLE is the history of IPI [19]. In our study, by analyzing these four subgroups of TLE patients with similar clinical characteristics, including the history of IPI, we could isolate the frequency of seizures as a main factor related to the GM atrophy. Despite the comparable percentage of patients with history of IPI, the gray matter damage was more pronounced in the groups of patients with frequent seizures.

Likewise, the duration of epilepsy was associated with extra-hippocampal GM atrophy in both TLE-HS and TLE-NL. As observed in the subgroups of patients with frequent seizures, thalamic atrophy was not associated with epilepsy duration. Interestingly, in TLE-HS, the most pronounced GM atrophy associated with epilepsy duration was observed in the anterior and lateral temporal lobes.

With these results, we hypothesize that the occurrence of HS is linked to the atrophy of structures connected to it, such as the thalamus, while other neocortical extra-temporal atrophy can be related to the occurrence of refractory seizures and to the evolution of the disease over time. The question that remains is whether the diffuse GM atrophy contributes to drug-resistance in TLE or these patients have more pronounced GM atrophy because of repeated seizures. Patients with new onset TLE should be more fully explored to better understand the role of seizures in structural abnormalities of TLE.

### Limitations of the study

As an MRI post-processing tool, there are some criticisms about the use of VBM [39]. However, although the use of VBM analysis in single subjects is still a challenge [39], this technique has proven to be reliable for group comparisons, with consistent findings in patients with TLE [22], [23]. Moreover, in the present study we used the most recent version of the VBM software (SPM8) together with the DARTEL algorithm. This combination allows the detection of more subtle abnormalities with less likelihood of false positive findings.

The classification of TLE patients as having frequent or infrequent seizures was based on a previous work [8]. Although the burden of infrequent seizures is subjective, the morbidity of epilepsy is certainly reduced in individuals with no GTCS and rare focal seizures. In fact, in our group we have combined patients with seizure remission (at least two years seizure free) and those with infrequent seizures. As these patients belong to a long-term cohort followed in our epilepsy center, we observe that the majority of these patients have long-term good seizure control. Furthermore, we believe that the dichotomy of seizure-free *versus* not seizure-free for this type of study is very artificial. Using this dichotomy, patients who had been seizure free for two years or longer and have a couple of seizures (often related to an external factor, such as acute illness, sleep deprivation, missed dose of AED, or during changes of AEDs because of side effects) and then are again seizure-free, would be lumped together with patients who have monthly or weekly seizures. The seemingly arbitrary limit of three seizures per year was based on the observation of our large cohort of patients with TLE and our previous studies [6], [8], because the great majority of our patients with well controlled TLE who present occasional breakthrough seizure and then go back to seizure control have up to three seizures in a 12-month period. Those who had been well controlled and then have more than three seizures in a 12-month period are more likely to become refractory to AEDs (this is usually related to tolerance and the honeymoon effect of AEDs). In fact, in the TLE-NL with infrequent seizures subgroup, six of 16 (62%) patients had been seizure-free for more than two years, five (12%) patients had only SPS, four (12%) patients had a single CPS and one (12%) had two CPS in the 12 months prior to evaluation; and in the TLE-HS with infrequent seizures subgroup, 10 of 16 (37%) had been seizure-free for more than two years, two (31%) patients had only SPS, two (25%) patients had a single CPS and two (6%) had two CPS in the 12-months prior to evaluation, showing that indeed these were overall patients with infrequent seizures.

In the present study, not all patients had an epileptogenic zone defined by ictal video-EEG recordings, but we believe this is expected in a large cohort of patients. Many of these patients were not candidates for surgery due to the mild course of their seizures. In this context, it would not be ethical to induce these individuals to undergo ictal recordings.

### Conclusions

According to our results, we propose that:

GM atrophy in the mesial structures in TLE-HS and in bilateral thalamus (anterior group) in both TLE-HS and TLE-NL are related to the original epileptogenic process;
GM atrophy in extra-temporal areas (in particular involving the frontal and parietal cortices) in both TLE-HS and TLE-NL are influenced by seizure frequency and epilepsy duration;
Neocortical GM atrophy in the temporal lobes is significantly influenced by the duration of epilepsy in TLE-HS.

The results of the present study add information to the possible mechanisms associated with the diffuse GM loss observed in TLE and reinforce the burden related to the occurrence of seizures in these patients. Further studies able to identify the time of onset of these structural abnormalities as well as the clinical relevance of their progression are necessary and might contribute to knowledge concerning epileptogenic mechanisms in TLE.

## Supporting Information

Table S1 Gray matter atrophy and volume increase in patients with MTLE-HS and MTLE-NL. Areas of gray matter atrophy and volume increase in patients with MTLE-HS and MTLE-NL detected by VBM analysis (*Two sample T-test: p<0.05, FWE, minimum of 30 voxels; **Two sample T-test: p<0.001, uncorrected, minimum of 30 voxels). MTLE-HS: mesial temporal lobe epilepsy with MRI signs of hippocampal sclerosis; MTLE-NL: mesial temporal lobe epilepsy with normal MRI; GM: gray matter; VBM: voxel based morphometry; FWE: family-wise error.
(DOCX)

Table S2 Clinical data of subgroups of MTLE-HS and MTLE-NL patients according to the AED response. * 2 had 2 CPS, 2 had a single CPS and 2 had only SPS in the 12 months prior to evaluation; ** 1 had 2 CPS, 4 had a single CPS and 5 had only SPS in the 12 months prior to evaluation. MTLE-HS: mesial temporal lobe epilepsy with MRI signs of hippocampal sclerosis; MTLE-NL: mesial temporal lobe epilepsy with normal MRI; good sz control: good seizure control; Ref: refractory; FS: febrile seizure; IPI: initial precipitating injury; AED: anti-epileptic drug; SGTCS: secondary generalized tonic-clonic seizures; CPC: complex partial seizure; SPS: simple partial seizure.
(DOCX)

Table S3 Gray matter atrophy in MTLE-HS and MTLE-NL with poor or good seizure control. Areas of gray matter atrophy and volume increase in patients with MTLE-HS and MTLE-NL detected by VBM analysis (Two sample T-test; MTLE-NL: p<0.001, uncorrected, minimum of 30 voxels). MTLE-HS: mesial temporal lobe epilepsy with MRI signs of hippocampal sclerosis; MTLE-NL: mesial temporal lobe epilepsy with normal MRI; GM: gray matter; VBM: voxel based morphometry; FWE: family-wise error.
(DOCX)

Table S4 Correlation between gray matter atrophy and epilepsy duration. Areas of gray matter atrophy correlated with epilepsy duration MTLE-HS and MTLE-NL patients as detected by VBM analysis (Multiple regression, p<0.001, uncorrected, minimum of 30 voxels). Only clusters with at least 500 contiguous voxels are described. MTLE-HS: mesial temporal lobe epilepsy with MRI signs of hippocampal sclerosis; MTLE-NL: mesial temporal lobe epilepsy with normal MRI; VBM: voxel based morphometry.
(DOCX)
