# Supplementary material for: Frequent Seizures Are Associated with a Network of Gray Matter Atrophy in Temporal Lobe Epilepsy with or without Hippocampal Sclerosis
Source: PLoS One. 2014 Jan 27;9(1):e85843. doi: 10.1371/journal.pone.0085843 (PMC3903486; doi:10.1371/journal.pone.0085843)
Supplement: Table S1 — Gray matter atrophy and volume increase in patients with MTLE-HS and MTLE-NL. Areas of gray matter atrophy and volume increase in patients with MTLE-HS and MTLE-NL detected by VBM analysis (*Two sample T-test: p<0.05, FWE, minimum of 30 voxels; **Two sample T-test: p<0.001, uncorrected, minimum of 30 voxels). MTLE-HS: mesial temporal lobe epilepsy with MRI signs of hippocampal sclerosis; MTLE-NL: mesial temporal lobe epilepsy with normal MRI; GM: gray matter; VBM: voxel based morphometry; FWE: family-wise error. (DOCX) [file pone.0085843.s001.docx]

**Table S1**: Gray matter atrophy and volume increase in patients with MTLE-HS and MTLE-NL

| **Groups: GM atrophy** | **Nº Voxel of the cluster** | **Area** | **Side** | **T score** | **MNI Coordinates** |
| --- | --- | --- | --- | --- | --- |
| **MTLE-HS*** | 8205 | Hippocampus | Left | 12.68 | -27 -21 -11 |
|  |  | Parahippocampal Gyrus | Left | 7.66 | -8 -36 3 |
|  |  | Thalamus | Left | 7.43 | -15 -27 1 |
|  | 1938 | Postcentral Gyrus (BA 3) | Left | 7.20 | -24 -28 66 |
|  | 1236 | Middle Temporal Gyrus (BA21) | Left | 6.74 | -38 5 -32 |
|  |  | Superior Temporal Gyrus (BA 38) | Left | 6.25 | -35 9 -24 |
|  | 1877 | Middle Occipital Gyrus (BA 19) | Left | 6.64 | -24 -93 15 |
|  |  | Cuneus (BA 19) | Left | 6.31 | -27 -88 27 |
|  |  | Precuneus (BA 19) | Left | 5.94 | -27 -72 40 |
|  | 526 | Precentral Gyrus (BA 4) | Right | 5.96 | 36 -19 52 |
|  | 86 | Cuneus (BA 7) | Left | 5.59 | -8 -73 30 |
|  |  | Cuneus (BA 17) | Right | 5.42 | 2 -85 7 |
|  | 32 | Caudate | Left | 5.29 | -5 15 6 |
| **MTLE-NL**** | 727 | Superior Frontal Gyrus (BA 11) | Left | 4.71 | -14 65 -15 |
|  | 755 | Precentral Gyrus (BA 6) | Right | 4.13 | 44 -12 30 |
|  | 394 | Postcentral Gyrus (BA 3) | Left | 3.95 | -27 -30 51 |
|  | 86 | Cuneus (BA 17) | Right | 3.63 | 3 -93 1 |
|  | 477 | Thalamus (Ventral Posterior Medial Nucleus) | Right | 3.62 | 15 -22 3 |
|  |  | Thalamus (Medial Dorsal Nucleus) | Left | 3.59 | -5 -13 9 |
|  |  | Thalamus (Pulvinar) | Right | 3.51 | 3 -27 7 |
|  | 66 | Medial Frontal Gyrus (BA 10) | Left | 3.46 | -5 62 7 |
|  | 35 | Middle Occipital Gyrus | Right | 3.41 | 29 -87 12 |
| **Groups: GM increase** | **Nº Voxel of the cluster** | **Area** | **Side** | **T score** | **MNI Coordinates** |
| **MTLE-HS**** | 164 | Pons | Right | 4.55 | 9 -37 -43 |
|  | 704 | Cingulate Gyrus | Right | 5.51 | 16 9 -31 |
|  | 117 | Uncus | Left | 3.93 | -13 6 -31 |
|  | 87 | Cerebellum, Posterior Lobe | Right | 3.50 | 54 -69 -22 |
| **MTLE-NL**** | 228 | Pons | Right | 4.27 | 6 -37 -42 |
|  | 48 | Inferior Temporal Gyrus | Right | 3.60 | 55 -22 -18 |
|  | 53 | Anterior Cingulate | Right | 3.90 | 1 1 -3 |

Areas of gray matter atrophy and volume increase in patients with MTLE-HS and MTLE-NL detected by VBM analysis (*Two sample T-test: p<0.05, FWE, minimum of 30 voxels; **Two sample T-test: p<0.001, uncorrected, minimum of 30 voxels). MTLE-HS: mesial temporal lobe epilepsy with MRI signs of hippocampal sclerosis; MTLE-NL: mesial temporal lobe epilepsy with normal MRI; GM: gray matter; VBM: voxel based morphometry; FWE: family-wise error.
