# Supplementary material for: Frequent Seizures Are Associated with a Network of Gray Matter Atrophy in Temporal Lobe Epilepsy with or without Hippocampal Sclerosis
Source: PLoS One. 2014 Jan 27;9(1):e85843. doi: 10.1371/journal.pone.0085843 (PMC3903486; doi:10.1371/journal.pone.0085843)
Supplement: Table S2 — Clinical data of subgroups of MTLE-HS and MTLE-NL patients according to the AED response. * 2 had 2 CPS, 2 had a single CPS and 2 had only SPS in the 12 months prior to evaluation; ** 1 had 2 CPS, 4 had a single CPS and 5 had only SPS in the 12 months prior to evaluation. MTLE-HS: mesial temporal lobe epilepsy with MRI signs of hippocampal sclerosis; MTLE-NL: mesial temporal lobe epilepsy with normal MRI; good sz control: good seizure control; Ref: refractory; FS: febrile seizure; IPI: initial precipitating injury; AED: anti-epileptic drug; SGTCS: secondary generalized tonic-clonic seizures; CPC: complex partial seizure; SPS: simple partial seizure. (DOCX) [file pone.0085843.s002.docx]

**Table S2**: Clinical data of subgroups of MTLE-HS and MTLE-NL patients according to the AED response

|  | **MTLE-NL with infrequent seizures (n=16)** | **MTLE-NL with frequent seizures (n=16)** | **MTLE-HS with infrequent seizures (n=16)** | **MTLE-HS with frequent seizures (n=16)** |
| --- | --- | --- | --- | --- |
| Sex | 7 female; 9 male | 7 female; 9 male | 7 female; 9 male | 7 female; 9 male |
| Age (range) | 41 years (19-74) | 42 years (20-55) | 46 years (23-61) | 42 years (26-62) |
| Age seizure onset(range) | 14 years (3-25) | 18 years (3-31) | 18 years (4-36) | 17 years (2-30) |
| Family history of epilepsy | 11 | 10 | 7 | 7 |
| FS/IPI | 2/7 | 1/4 | 1/6 | 1/6 |
| SE | zero | zero | zero | zero |
| Duration of epilepsy (range) | 24 years (3-48) | 23 years (7-50) | 25 years (2-54) | 26 years (9-45) |
| Time of active epilepsy (range) | 19 years (3-38) | 20 years (4-47) | 21 years (2-41) | 23 years (9-39) |
| Laterality of epileptogenic zone | 4 Right; 12 Left | 4 Right; 12 Left | 4 Right; 12 Left | 4 Right; 12 Left |
| Patients with GTCS in the previous year | 1 | 2 | zero | 1 |
| Seizure remission (>2 years) | 10 | zero | 6 | zero |

* 2 had 2 CPS, 2 had a single CPS and 2 had only SPS in the 12 months prior to evaluation; ** 1 had 2 CPS, 4 had a single CPS and 5 had only SPS in the 12 months prior to evaluation.

MTLE-HS: mesial temporal lobe epilepsy with MRI signs of hippocampal sclerosis; MTLE-NL: mesial temporal lobe epilepsy with normal MRI; good sz control: good seizure control; Ref: refractory; FS: febrile seizure; IPI: initial precipitating injury; AED: anti-epileptic drug; SGTCS: secondary generalized tonic-clonic seizures; CPC: complex partial seizure; SPS: simple partial seizure.
