# Supplementary material for: Frequent Seizures Are Associated with a Network of Gray Matter Atrophy in Temporal Lobe Epilepsy with or without Hippocampal Sclerosis
Source: PLoS One. 2014 Jan 27;9(1):e85843. doi: 10.1371/journal.pone.0085843 (PMC3903486; doi:10.1371/journal.pone.0085843)
Supplement: Table S3 — Gray matter atrophy in MTLE-HS and MTLE-NL with poor or good seizure control. Areas of gray matter atrophy and volume increase in patients with MTLE-HS and MTLE-NL detected by VBM analysis (Two sample T-test; MTLE-NL: p<0.001, uncorrected, minimum of 30 voxels). MTLE-HS: mesial temporal lobe epilepsy with MRI signs of hippocampal sclerosis; MTLE-NL: mesial temporal lobe epilepsy with normal MRI; GM: gray matter; VBM: voxel based morphometry; FWE: family-wise error. (DOCX) [file pone.0085843.s003.docx]

**Table S3**: Gray matter atrophy in MTLE-HS and MTLE-NL with poor or good seizure control

| **Groups: GM atrophy** | **Nº Voxel of the cluster** | **Area** | **Side** | **T Score** | **MNI Coordinates** |
| --- | --- | --- | --- | --- | --- |
| **MTLE-HS with frequent seizures** | 6130 | Hippocampus | Left | 9.33 | -30 -15 -17 |
|  |  | Thalamus (Medial Dorsal Nucleus) | Left | 5.14 | -5 -16 6 |
|  | 1875 | Superior Temporal Gyrus (BA 38) | Left | 5.23 | -36 12 -23 |
|  |  | Middle Temporal Gyrus (BA 21) | Left | 5.04 | -36 5 -33 |
|  | 202 | Superior Parietal Lobule (BA 5) | Right | 4.88 | 20 -43 60 |
|  | 2383 | Medial Frontal Gyrus (BA 9) | Left | 4.42 | 0 50 18 |
|  |  | Superior Frontal Gyrus (BA 8) | Left | 4.20 | -24 27 49 |
|  | 284 | Caudate | Left | 4.27 | -6 17 7 |
|  | 419 | Inferior Frontal Gyrus (BA 10) | Left | 4.10 | -47 45 0 |
|  | 730 | Inferior Parietal Lobule (BA 40) | Right | 4.09 | 51 -27 46 |
|  |  | Precentral Gyrus (BA 4) | Right | 4.08 | 39 -19 54 |
|  | 139 | Middle Frontal Gyrus (BA 10) | Left | 3.80 | -33 48 6 |
|  | 188 | Postcentral Gyrus (BA 5) | Left | 3.79 | -21 -42 63 |
|  | 81 | Precentral Gyrus (BA 4) | Left | 3.36 | -48 -13 42 |
|  | 42 | Precuneus (BA 7) | Right | 3.59 | 12 -5 49 |
| **MTLE-HS with infrequent seizures** | 7519 | Hippocampus | Left | 8.55 | -26 -22 -11 |
|  |  | Thalamus (Medial Dorsal Nucleus) | Left | 5.45 | -3 -15 9 |
|  | 1941 | Caudate | Left | 5.24 | -8 17 7 |
|  | 1568 | Cerebellum, Posterior Lobe | Left | 4.94 | -21 -75 -44 |
|  | 694 | Precentral Gyrus (BA 4) | Right | 4.35 | 32 -25 64 |
|  | 83 | Parahippocampal Gyrus | Right | 4.07 | 23 -25 -6 |
|  | 36 | Subcallosal Gyrus (BA 25) | Right | 3.33 | 12 25 -9 |
| **MTLE-NL with frequent seizures** | 296 | Precentral Gyrus (BA 4) | Right | 4.25 | 33 -27 64 |
|  | 210 | Precentral Gyrus (BA 6) | Left | 3.81 | -36 -18 67 |
|  | 189 | Thalamus | Right | 3.70 | 2 -25 10 |
|  | 306 | Superior Frontal Gyrus | Left | 3.70 | -21 62 -2 |
|  | 75 | Postcentral Gyrus (BA 3) | Right | 3.64 | 41 -19 48 |
|  | 68 | Precuneus (BA 19) | Left | 3.63 | -33 -84 42 |
|  | 49 | Middle Temporal Gyrus (BA 21) | Left | 3.63 | -41 5 -30 |
|  | 30 | Caudate | Left | 3.50 | -23 -27 64 |
|  |  |  |  |  |  |

Areas of gray matter atrophy and volume increase in patients with MTLE-HS and MTLE-NL detected by VBM analysis (Two sample T-test; MTLE-NL: p<0.001, uncorrected, minimum of 30 voxels). MTLE-HS: mesial temporal lobe epilepsy with MRI signs of hippocampal sclerosis; MTLE-NL: mesial temporal lobe epilepsy with normal MRI; GM: gray matter; VBM: voxel based morphometry; FWE: family-wise error.
