# Supplementary material for: Frequent Seizures Are Associated with a Network of Gray Matter Atrophy in Temporal Lobe Epilepsy with or without Hippocampal Sclerosis
Source: PLoS One. 2014 Jan 27;9(1):e85843. doi: 10.1371/journal.pone.0085843 (PMC3903486; doi:10.1371/journal.pone.0085843)
Supplement: Table S4 — Correlation between gray matter atrophy and epilepsy duration. Areas of gray matter atrophy correlated with epilepsy duration MTLE-HS and MTLE-NL patients as detected by VBM analysis (Multiple regression, p<0.001, uncorrected, minimum of 30 voxels). Only clusters with at least 500 contiguous voxels are described. MTLE-HS: mesial temporal lobe epilepsy with MRI signs of hippocampal sclerosis; MTLE-NL: mesial temporal lobe epilepsy with normal MRI; VBM: voxel based morphometry. (DOCX) [file pone.0085843.s004.docx]

**Table S4:** Correlation between gray matter atrophy and epilepsy duration

|  | **Nº Voxel of the cluster** | **Area** | **Side** | **T score** | **MNI Coordinates** |
| --- | --- | --- | --- | --- | --- |
| **MTLE-HS** | 13329 | Superior Temporal Gyrus (BA 22) | Right | 5.90 | 59, -4, 3 |
|  |  | Inferior Frontal Gyrus (BA 9) | Right | 5.85 | 51, 14, 28 |
|  | 7783 | Middle Temporal Gyrus (BA 21) | Left | 5.72 | -44, -3, -17 |
|  |  | Superior Temporal Gyrus (BA 38) | Left | 5.06 | -33, 8, -23 |
|  | 3451 | Cerebellum, Posterior Lobe | Left | 4.80 | -29, -45, -41 |
|  | 4634 | Cingulate Gyrus (BA 32) | Right | 4.78 | 2, 36, 27 |
|  |  | Medial Frontal Gyrus (BA 8) | Right | 4.92 | 5, 51, 39 |
|  |  | Superior Frontal Gyrus (BA 8) | Right | 4.61 | 3, 38, 45 |
|  | 597 | Cerebellum, Posterior Lobe | Right | 4.69 | 30, -42, -42 |
|  | 1057 | Inferior Temporal Gyrus (BA 20) | Left | 4.17 | -58, -40, -20 |
|  | 979 | Superior Frontal Gyrus | Left | 4.56 | -27, 60, -8 |
|  | 1425 | Precentral Gyrus | Left | 4.51 | -47, -7, 42 |
|  |  | Middle Frontal Gyrus | Left | 4.05 | -51, 5, 43 |
|  | 1420 | Cingulate Gyrus | Left | 4.17 | -3, -27, 33 |
|  | 1059 | Insula | Right | 3.95 | 51, -39, 18 |
|  |  | Inferior Parietal Lobule (BA 40) | Right | 3.77 | 56, -49, 39 |
|  | 829 | Medial Frontal Gyrus | Left | 5.52 | -6, 56, -15 |
| **MTLE-NL** | 899 | Middle Frontal Gyrus (BA 6) | Right | 5.00 | 41, 0, 43 |
|  | 3792 | Cerebellum, Anterior Lobe | Right | 5.36 | 9, -43, -8 |
|  |  | Cerebellum, Posterior Lobe | Left | 5.33 | -9, -61, -12 |
|  | 847 | Insula | Right | 4.27 | 47, 0, 4 |

Areas of gray matter atrophy correlated with epilepsy duration MTLE-HS and MTLE-NL patients as detected by VBM analysis (Multiple regression, p<0.001, uncorrected, minimum of 30 voxels). Only clusters with at least 500 contiguous voxels are described. MTLE-HS: mesial temporal lobe epilepsy with MRI signs of hippocampal sclerosis; MTLE-NL: mesial temporal lobe epilepsy with normal MRI; VBM: voxel based morphometry.
